# Supplementary material for: A Strategy for Gene Knockdown in Dinoflagellates
Source: Microorganisms. 2022 May 31;10(6):1131. doi: 10.3390/microorganisms10061131 (PMC9228228; doi:10.3390/microorganisms10061131)
Supplement: Supplementary file 1 [file microorganisms-10-01131-s001.zip › microorganisms-1687113-supplementary.pdf]

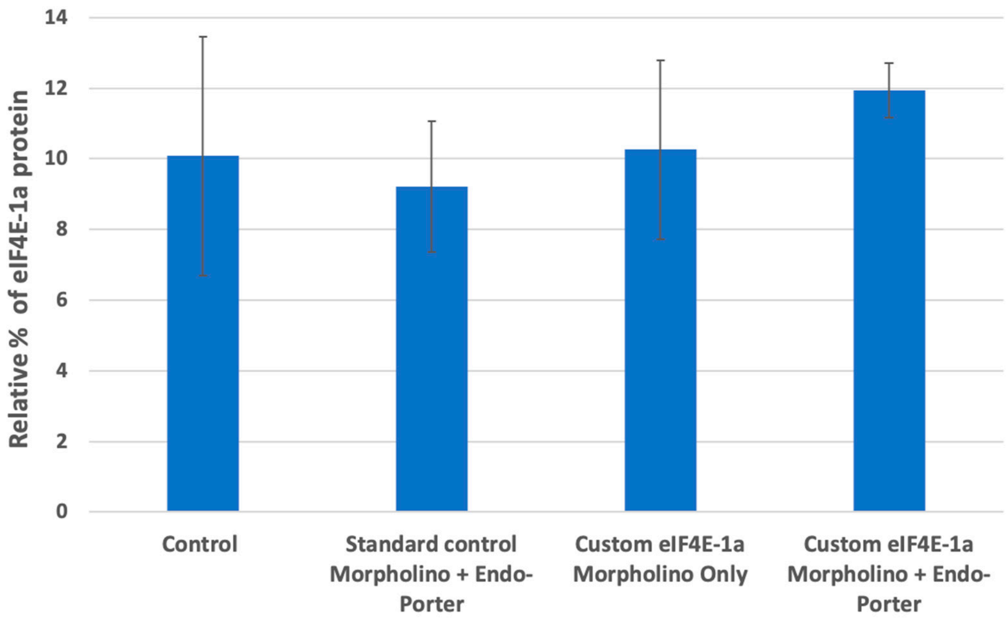

Supplemental Figure S1. Western blot analyses for eIF4E-1a concentrations within control and treated cells at 96 hours post-MO [10 μM] addition (N = 3). Protein loading and relative expression levels was verified by probing with anti-eIF4E-1a mouse monoclonal and HRP-conjugated anti-mouse IgG and compared to total protein volumes. The effect of the custom translation-blocking morpholinos show no effect after 96 hours of treatment. Statistical analysis was done with t-tests using pooled standard deviations.

No significant difference between any samples found based on a *p*-value of < 0.05.

Supplemental Table S1. Lissamine uptake in *A. carterae* cells 48 hours post treatment. Intensity of the Lissamine signal is shown in relative fluorescence units (RFUs). A large positive-skew is evident in the "Antisense Morpholino + Endo Porter" culture, characterized by the extreme difference in the mean and median.

Condition	Mean (RFUs)	Median (RFUs)
Control	3043.46	2143.67
Endo Porter Only	2572.00	2296.67
Antisense Morpholino Only	3706.23	2209.00
Antisense Morpholino + Endo Porter	153,246.42	3236.17
